# Editorial: Contextualizing interviews to detect verbal cues to truths and deceit

**DOI:** 10.3389/fpsyg.2023.1300160

**Published:** 2023-10-10

**Authors:** Haneen Deeb, Jacqueline R. Evans, Aldert Vrij

**Affiliations:** ^1^Department of Psychology, University of Portsmouth, Portsmouth, United Kingdom; ^2^Department of Psychology, Florida International University, Miami, FL, United States

**Keywords:** lie detection, deception, verbal cues, context, interviews

Lie detection in forensic interviews is often based on verbal cues, non-verbal cues, or a combination of verbal and non-verbal cues. Scientific evidence unveiled that verbal cues are more diagnostic than non-verbal cues (DePaulo et al., 2003), so research in the field has mostly shifted focus from non-verbal to verbal lie detection (Vrij et al., 2019). The majority of the tested verbal cues are cues to truthfulness. For example, it has been established that truth tellers provide more detailed (Amado et al., 2016), plausible (Vrij et al., 2021a), and verifiable information (Palena et al., 2021) than lie tellers. However, verbal cues to deceit (i.e., those which occur more among lie tellers than truth tellers), are rarely examined. This is important because practitioners look for cues that are present rather than cues that are absent (Vrij et al., 2023). For example, it is easier to look at the presence of justifications than at the absence of justifications when assessing suspect veracity. That said, there are some cues to deceit that have been examined, including common knowledge details and self-handicapping strategies (Vrij et al., 2021b) and cognitive processes (Masip et al., 2005). Thus, one aim of this Research Topic was to encourage the testing of more verbal cues, and ideally to identify more verbal cues to deceit.

Caso et al. experimentally examined verbal cues to truthfulness and deceit across different lie types. Italian participants said the truth or provided an outright or embedded lie about a past experience. Truthful accounts included significantly more complications than outright—but not embedded—lies which contrasted with previous findings in the United Kingdom (UK; Verigin et al., 2020).

Dunbar et al. examined cues to truthfulness and deceit in job interviews. The experiment was run online in groups of 4 or 5. Participants read the profile of one of five candidates and then presented a summary of the profile to the group for deliberation. Two participants were allocated to be deceivers and were given a low quality resume that they had to recommend. Truth tellers were given a high or a medium quality resume. Deceivers' speech was more complex than that of truth tellers. Further, when detected, deceivers were perceived as more untrustworthy than truth tellers.

Verbal cues cannot be isolated from context as some cues can be diagnostic in certain contexts but not in others (Markowitz and Hancock, 2022). Thus, another aim of this Research Topic was to understand the diagnosticity of verbal cues in different contexts. Given that the existing literature has tested samples in Western countries (e.g., the United States and the UK; Denault et al., 2022), some of the contributions in this issue were from countries/cultures which were rarely—if ever—tested.

In two experiments, Tache et al. examined verbal cues to truthfulness and deceit in individualistic and collectivistic cultures in the UK. Participants responded to expected and unexpected questions about an intended trip (Experiment 1) or to a sketch and timeline request about a past event (Experiment 2). Cultural differences but not veracity differences emerged in both experiments implying caution when generalizing across cultures.

Verbal cues can differ depending on the interviewee's language (Taylor et al., 2014). Thus, Dando et al. examined verbal cues of British and South Asian participants who spoke in their first or second language. The findings largely converged with previous research with a lie bias emerging when judging non-native speakers.

Instead of looking at cross-cultural contexts, Bagnall et al. looked at clinical differences between autistic and non-autistic adults who lied or told the truth about a virtual burglary scenario. Autistic and non-autistic truth tellers differed on extricating (verifiable) information but not on investigation-relevant information and statement-evidence consistency suggesting commonalities between the clinical samples.

Sergi et al. tested differences between truth tellers and lie tellers on individual characteristics (memory and impulsiveness) and Reality Monitoring verbal cues (realism, clarity, reconstructability). Self-reported poor memory and impulsivity were associated with more lying. Also, truth tellers' stories sounded more realistic, clear, and reconstructive than those of lie tellers.

Dykstra et al. examined verbal cues among maltreated and non-maltreated children. Children were coached to either conceal (lie tellers) or not (truth tellers) a transgression. More first-person plural pronouns and cognitive mechanism terms and less syntactically complex reports were diagnostic of lie telling. Maltreated children used more affect and negation terms and fewer words and complex statements than non-maltreated children but the two groups did not differ on veracity cues.

Rather than examining verbal cues, Zanette et al. asked judges to assess the veracity of children's statements according to race (Black vs. White). Participants in a crowdsourcing platform viewed a vignette and photo of a White or Black child who was interviewed about a transgression. White children were rated as lie tellers more than Black children which suggested a truth bias toward Black children. Internal motivation to not appear prejudiced, especially among White adults, moderated these effects.

The Research Topic also includes two survey studies that examined meta-cognitive processes in different contexts. In one of the studies, Tabata and Vrij asked Japanese participants an open question on strategies they use when lying. The self-reports resulted in 13 strategies which largely converged with previous findings with different samples. In another study, Junger et al. examined perceptions of (near) victims of fraud on how to reduce fraud victimization. For near victims of fraud, knowledge about fraud reduced victimization approximately half of the time. Actual victims of fraud self-reported that had they sought more information or paid more attention, victimization may have been prevented. Higher proportion of near victims than of actual victims suggested a lie bias in fraud settings.

In two review and opinion papers, Markowitz et al. and Levine argued that context matters as much as—if not more than—verbal cues. The two papers, however, differed in how they framed the utility of context for facilitating lie detection. Markowitz et al. extended their original “Contextual Organization of Language and Deception (COLD) framework” to explain contextual aspects (namely psychological dynamics, pragmatic goals, genre conventions, individual differences, situational opportunities, and interpersonal characteristics) that affect deceptive language and verbal cues. The authors recommended incorporating these aspects in research designs. In his opinion paper, Levine reasoned that lie detection should be based on content (e.g., background knowledge of the information that is being assessed, interview dynamics, etc.) rather than on cues or demeanor, because knowing content leads to more appropriate questioning and thus to better assessments.

The issue also features a bibliometric review of the research on investigative interviews. Denault and Talwar first provided a rich account of the history of coercive criminal interrogations and their evolution to ethical investigative interviews. This was followed by a listing of the top: journals, academic institutions, countries in which the research is published, research areas, publishing authors, keywords, and cited articles in the field. The authors then critically reviewed the context.

In conclusion, the issue showed that context is important and that there are times when verbal cues vary across contexts. However, other papers demonstrated that some verbal cues can be diagnostic across certain contexts. Thus, while different samples exhibit deception differently, researchers and practitioners can still look at stable cues and build on them when developing novel research or when assessing veracity.

## Author contributions

HD: Conceptualization, Writing—original draft, Writing—review and editing. JE: Conceptualization, Writing—review and editing. AV: Conceptualization, Writing—review and editing.
